# Integrated Analysis of the lncRNA-Associated ceRNA Network in Wilms Tumor via TARGET and GEO Databases

**DOI:** 10.1155/2022/2365991

**Published:** 2022-08-31

**Authors:** Biao An, Yuan Hu, Xiao Liang

**Affiliations:** Department of Gynecology and Obstetrics, Development and Related Disease of Women and Children Key Laboratory of Sichuan Province, Key Laboratory of Birth Defects and Related Diseases of Women and Children, Ministry of Education, West China Second Hospital, Sichuan University, Chengdu, China

## Abstract

Wilms tumor (WT) is the most common genitourinary renal tumor that typically occurs in children under 15 and is thought to be linked to somatic and germline mutations. However, the specific functional role of competing endogenous RNAs (ceRNAs) and their potential implications in WT remain unclear. In this study, we developed an lncRNA-mediated (long noncoding RNA-mediated) ceRNA network via the R packages for WT with expression data obtained from the tumor alterations relevant for genomics-driven therapy (TARGET) database. Unsupervised hierarchical clustering analysis revealed that the WT specimens could be clearly distinguished from healthy specimens with respect to the expression of disordered RNAs. A total of 1,607 differentially expressed (DE) lncRNAs, 116 DE microRNAs (DEmiRNAs), and 3,262 DE messenger RNAs (DEmRNAs) were identified as WT-specific RNAs, and a lncRNA-miRNA-mRNA ceRNA network with 159 DElncRNAs, 18 DEmiRNAs, 131 DEmRNAs, and 792 interactions was constructed. According to the clinical survival data, 12 DElncRNAs, 5 DEmRNAs, and 2 DEmiRNAs were selected from the ceRNA network that could significantly impact the overall survival of WT patients (*P* < 0.05). Functional enrichment analysis showed that the biological processes and pathways of DEmRNAs, such as cell cycle and virus infection, may be associated with WT. The present study constructed a dysregulated lncRNA-mediated ceRNA network in WT and discovered that lncRNA-mediated ceRNAs may serve as important regulators in WT development and progression. Survival-associated RNAs may serve as new potential biomarkers, suggesting that the constructed ceRNA network in WT might be important for determining optimal therapeutic strategies.

## 1. Introduction

Wilms tumor (WT) is the most common genitourinary renal tumor that typically occurs in early childhood [1], which is usually found in one or both kidneys and may metastasize to other important organs [2]. With new medicines and innovative immune therapies performed in WT [3], the survival rate of WT has remarkably increased [4]. However, as a malignant tumor mainly occurs in young children, a deep understanding of the occurrence and development of WT still requires more attention.

With the development of sequencing technology, increased dysregulation and mutations of DNA or RNA in human carcinoma including epigenetic alterations [5] were revealed, and the bioinformatics analysis showed the important roles they played in many tumors. However, the knowledge of the genetic underpinnings of WT was largely limited to somatic and germline mutations [6], such as aberrations of tumor protein 53 (TP53), the WT gene on the X chromosome (WTX), WT gene 1 (WT1), catenin beta 1 (CTNNB1), and the imprinted 11p15 region [7–9].

The competing endogenous RNA (ceRNA) hypothesis was first presented in 2011, which postulated that RNAs communicate with each other through shared microRNA (miRNA) response elements (MREs) [10]. Since a miRNA modulates the expression of several target messenger RNAs (mRNAs), each mRNA may be regulated by multiple miRNAs [11]. mRNAs, long noncoding RNAs (lncRNAs), and other RNA transcripts, which can act as endogenous miRNA sponges, in the ceRNA network can form a large-scale regulatory network. The dysregulation of RNAs involved in the ceRNA network has been previously studied in many cancers [12, 13]. The key RNAs are regarded as cancer biomarkers and contribute to tumor progression [14–16].

Wilms tumor is relatively rare in absolute numbers (1 in 10,000 children). The scarcity of WT specimen makes it difficult to perform experimental research on WT. Moreover, the commercially available, previously widely used purported WT cell lines, including WT-CLS1 [17], G401 [18, 19], and SK-NEP-1 [19, 20], turned out to be misclassified. Thus, the studies about WT are limited by the paucity of available cell/tissue specimens, and bioinformatics analysis becomes an effective and economical strategy for WT study.

To the best of our knowledge, this study is the first time to construct a lncRNA-mediated WT-specific ceRNA network with both GEO and TARGET databases. In the present study, we identified WT-specific miRNAs and mRNAs convergence in both GEO and TARGET databases and provide novel insight into a better understanding of lncRNA-mediated ceRNA regulation in the tumorigenesis and progression of WT. We hope this work helps elucidate the lncRNA-miRNA-mRNA crosstalk in WT and provides further insight into its molecular mechanisms.

## 2. Materials and Methods

### 2.1. Data Acquisition and Processing

**Figure 1:**
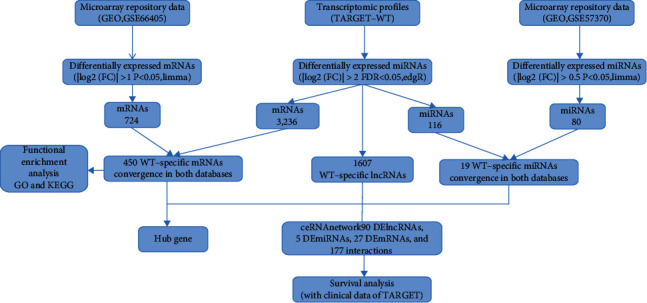
Flow diagram of the study.

All datasets used in the present study were downloaded from the GEO database (https://www.ncbi.nlm.nih.gov/geo/) and the TARGET database (https://ocg.cancer.gov/programs/target). In the GSE66405 dataset, mRNA expression of 28 WT and 4 normal kidney samples were determined using the Agilent-039494 SurePrint G3 Human GE v2 8 × 60 K (GPL17077) platform. In the GSE57370 dataset, miRNA expression of 62 WT and 4 normal kidney samples were determined with the Agilent-031181 Unrestricted_Human_miRNA_V16.0_Microarray (GPL16770) platform. The expression quantification of mRNAs, lncRNAs, and miRNAs was obtained from the TARGET database. 125 WT patients with corresponding clinical data and 6 healthy controls were enrolled. The main characteristics of patients enrolled in the study are shown in Table 1, and the study flow diagram is shown in Figure 1.

### 2.2. Identification of DERNAs between WT Tissues and Healthy Tissues

The R 3.6.0 software (https://www.r-project.org/) was used to identify the differentially expressed RNAs between WT and normal samples. In the GSE66405 dataset, differentially expressed mRNAs were identified using the “limma” package with |log_2_ fold change (FC)| >1 and *P* < 0.05 as cutoff criteria. Similarly, differentially expressed miRNAs were screened with |log_2_ FC| > 0.5 and *P* < 0.05 in GSE57370. Additionally, the differentially expressed RNAs in the TARGET dataset were determined using the “edgeR” package with |log_2_ FC| > 2 and a false discovery rate (FDR) < 0.01 as the cutoff criteria.

### 2.3. Functional Enrichment Analysis

Functional enrichment analysis was performed for the WT-specific mRNAs involved in the ceRNA network to understand the underlying functional implications of these mRNAs in Wilms tumor. Gene ontology (GO) functional enrichment analysis and Kyoto Encyclopedia of Genes and Genomes (KEGG) pathway enrichment analysis were conducted using the R package “clusterProfiler” [21]. Disease ontology (DO) analysis was conducted using the R package “DOSE” [22]. *P* < 0.05 was set as the statistical significance threshold criterion for enrichment analysis.

### 2.4. Construction of the Protein-Protein Interaction Network

mRNAs convergence in TARGET and GSE66405 were determined as WT-specific mRNAs. The direct and indirect correlations between WT-specific mRNAs were assessed from the Search Tool for the Retrieval of Interacting Genes/Proteins (STRING; Version 11.0; http://string-db.org/) database (with the minimum required interaction score of 0.9) [23]. The PPI network was reconstructed via Cytoscape software [24] (Version 3.8.0). Functionally related clusters were further identified with the molecular complex detection algorithm (MCODE; Version: 1.5.1), based on topology to locate densely connected regions. The mRNAs in the top cluster selected were taken as hub genes.

### 2.5. Expression Validation of Hub Genes in Oncomine Database

The Cutcliffe renal dataset and Yusenko renal dataset in Oncomine (https://www.Oncomine.org) were used to verify the differential expression of hub genes. In the Cutcliffe renal dataset, mRNA expression of 18 WT and 3 fetal kidneys were determined using human genome U133 A array in which ASPM and NUF2 were not tested. In the Yusenko renal dataset, mRNA expression of 4 WT and 2 fetal kidneys was determined using human genome U133 Plus 2.0 array. The log2 median-centered intensity value was visualized with GraphPad Prism (Version 5.0).

### 2.6. Construction of the ceRNA Network

The interactions between WT-specific lncRNAs and WT-specific miRNAs were predicted using the miRcode database (http://www.mircode.org/) [25]. Then, WT-specific miRNAs were modified with the StarBase v2.0 database (http://starbase. sysu.edu.cn/), and their target mRNAs were retrieved according to three different databases (TargetScan [26], miRTarBase [27], and miRDB [28]). Only mRNAs predicted by all three databases were considered as candidate DEmRNAs for further study. Then, the lncRNA-miRNA-mRNA ceRNA network was constructed and visualized using the Cytoscape software (Version 3.8.0) [29].

### 2.7. Survival Analysis

WT patients were divided into a specific RNA high-expressing group and a specific RNA low-expressing group, with the median expression value as the cutoff value. Survival analysis between the specific RNA high-expressing and specific RNA low-expressing groups was evaluated using the Kaplan–Meier survival curve and log-rank test analysis. The survival-associated DERNAs (*P* < 0.05) were identified as prognosis-associated RNAs.

## 3. Results

### 3.1. Identification of WT-Specific mRNAs

**Figure 2:**
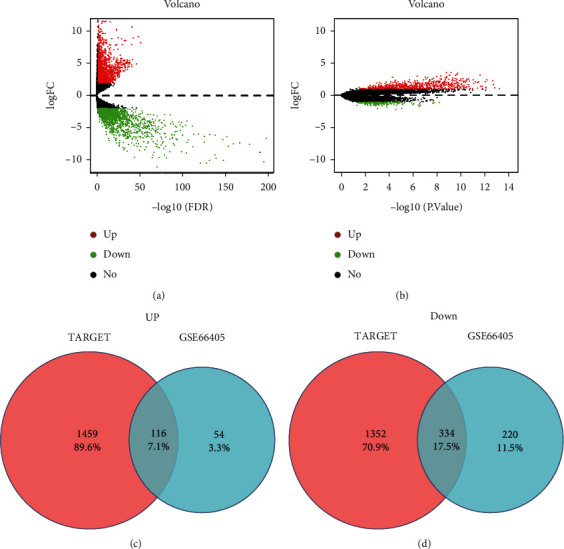
Identification of WT-specific mRNAs. Differentially expressed mRNA in TARGET (a) and GSE66405 (b) with the aforementioned cutoff thresholds is depicted in the volcano map. The red/green points represent the upregulation/downregulation of mRNAs, and the black points represent RNAs with no significant difference. The 116 upregulated (c) and 334 downregulated (d) WT-specific mRNAs convergence in both databases is presented in Venn diagrams

A total of differentially expressed 3,262 differentially expressed mRNAs (1,756 upregulated and 1,506 downregulated) were altered significantly in the TARGET-WT dataset. In addition, 724 (170 upregulated and 554 downregulated) differentially expressed mRNAs in GSE66405 were determined with the aforementioned cutoff thresholds. The distribution of all differentially expressed mRNAs is depicted in the volcano maps shown in Figures 2(a) and 2(b). The 116 upregulated and 334 downregulated mRNAs convergence in both databases were identified as WT-specific mRNAs and presented in Venn diagrams (Figures 2(c) and 2(d)).

### 3.2. Functional Enrichment Analysis

**Figure 3:**
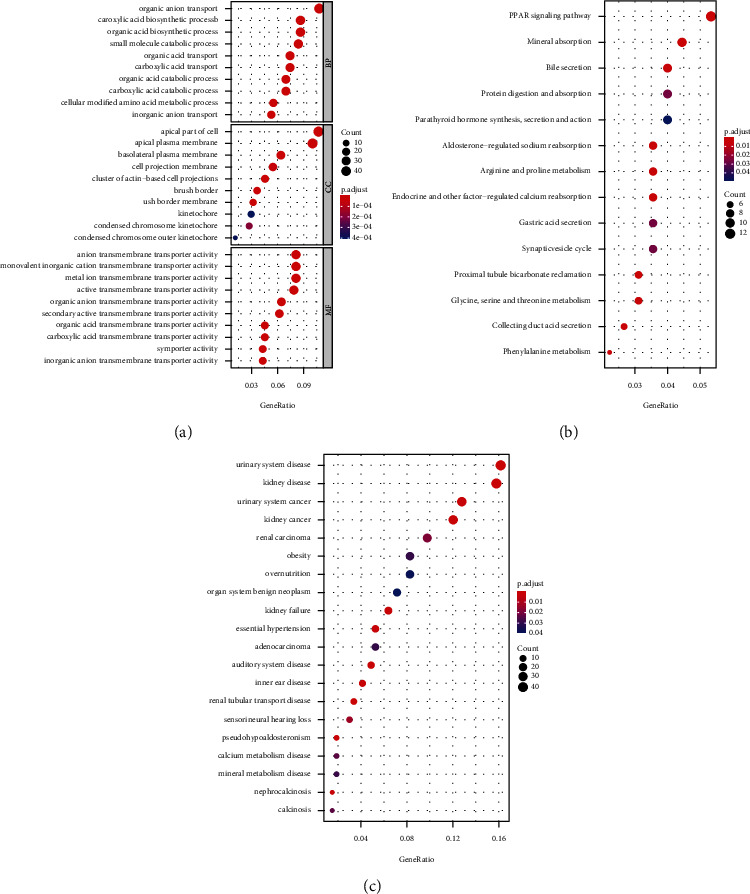
Functional enrichment analysis for WT-specific mRNAs. (a) The top 10 predominant GO.BP, GO.CC, and GO.MF terms are shown in the dot plot. (b) All the 14 significantly enriched KEGG pathways are shown in the dot plot. (c) The DO enrichment analysis showed the top 20 WT-specific mRNAs associated with diseases.

To outline the potential function of the WT-specific mRNAs, functional enrichment analysis was performed with “ClusterProfiler” and “DOSE.” Functional enrichment analysis revealed that a total of 394 GO terms, including 277 biological process terms (GO.BP), 31 cellular component terms (GO.CC), and 86 molecular function terms (GO.MF), were enriched. The top 10 predominant BP terms, CC terms, and MF terms in GO functional enrichment analysis are shown in Figure 3(a) which were concerned with organic acid carboxylic and transport. These results suggested that tumorigenesis and the development of WT may be related to the dysfunction of renal ion transport.

14 significantly enriched KEGG pathways were identified and are shown in Figure 3(b). KEGG results showed that the “PPAR signaling pathway” was the most concerned pathway in WT. PPARs belong to the family of ligand-activated nuclear receptors. Specific PPAR ligands have been proposed as potential therapies for a variety of diseases such as metabolic syndrome and cancer [30]. Moreover, DO analysis (Figure 3(c)) showed that WT was not only a urinary system disease but also concerned with “nephrocalcinosis” and calcium/mineral metabolism disease, which were verified in GO and KEGG analyses. Moreover, obesity and overnutrition were also involved, which may originate partly in lifestyle, in particular via markedly reduced levels of physical activity after diagnosis.

### 3.3. Protein-Protein Interaction Network and Hub Genes

**Figure 4:**
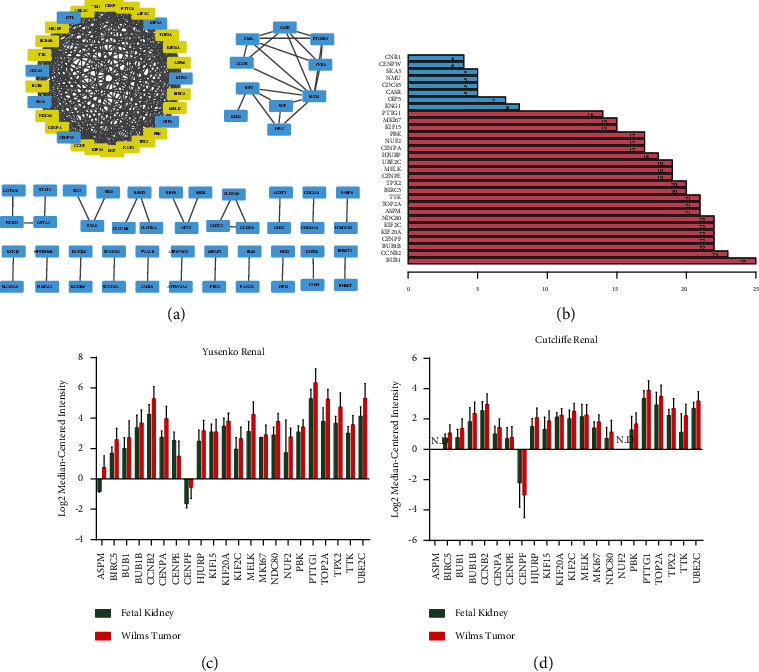
Identification and expression validation of hub genes. (a) The PPI network of WT-specific mRNAs shown by Cytoscape. (b) The degree distribution of the PPI network of the top 30 WT-specific mRNAs. (c) Expression validation of the hub genes performed with the Yusenko renal dataset. Mean ± SEM. (d) Expression validation of the hub genes performed with the Cutcliffe renal dataset. Mean ± SEM.

To better understand the interplay among the WT-specific mRNAs, a PPI network with 83 nodes and 276 edges was reconstructed by Cytoscape (Figure 4(a)). The degree distribution of the nodes in this PPI network was analyzed, and the mRNAs with top 30° are shown in Figure 4(b). BUB1 mitotic checkpoint serine (BUB1) was identified to have the highest degree in the overall network (degree = 25).

Based on the topology to locate densely connected regions, some genes are notably concentrated. With the help of MCODE, a hub gene cluster with 22 nodes and 203 edges was selected. These 22 hub genes were all upregulated and with a high degree in the overall network (Figure 4(b)). NUF2 (NUF2) was identified to be the seed gene of the cluster (degree = 14). Most of the hub genes were predominantly involved in the cell cycle (BUB1, mitotic checkpoint serine/threonine kinase B (BUB1B), cyclin B2 (CCNB2), and pituitary tumor-transforming 1 (PTTG1)) and oocyte meiosis pathways (BUB1, CCNB2, and PTTG1). Expression validation of the hub genes was performed with the Yusenko renal dataset (Figure 4(c)) and Cutcliffe renal dataset (Figure 4(d)) in Oncomine. Except for CENPF (centromere protein F) was unexpectedly downregulated, all the other hub genes were upregulated in WT tissues in both Oncomine datasets as expected.

### 3.4. Identification of WT-Specific miRNAs and lncRNAs

**Figure 5:**
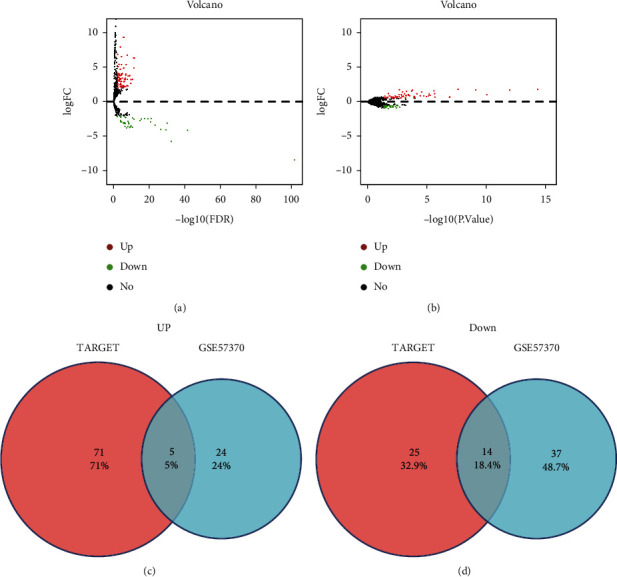
Identification of WT-specific miRNAs. Differentially expressed miRNA in TARGET (a) and GSE57370 (b) with the aforementioned cutoff thresholds was depicted in the volcano map. The red/green points represent the upregulation/downregulation of mRNAs, and the black points represent RNAs with no significant difference. the 5 upregulated (c) and 14 downregulated (d) WT-specific miRNAs convergence in both databases is presented in Venn diagrams.

A total of 116 differentially expressed miRNAs (including 77 upregulated and 39 downregulated) were identified as altered significantly in the TARGET-WT dataset and 80 (29 upregulated and 51 downregulated) differentially expressed miRNAs in GSE57370 were determined with aforementioned cutoff thresholds. The distribution of all differentially expressed miRNAs is depicted in the volcano maps shown in Figures 5(a) and 5(b). The 19 miRNAs convergence in both databases were identified as WT-specific miRNAs and are presented in Venn diagrams (Figures 5(c) and 5(d)).

### 3.5. ceRNA Network Construction in WT

**Figure 6:**
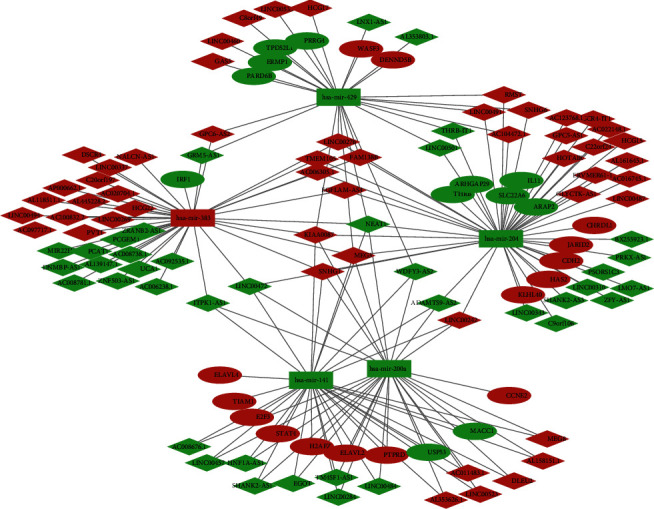
Construction of the ceRNA network.

1607 differentially expressed lncRNAs (including 851 upregulated and 756 downregulated) identified in TARGET database with the aforementioned cutoff thresholds were taken as WT-specific lncRNAs. Then, we screened the miRcode database with the WT-specific lncRNAs and obtained 689 interactions between 90 WT-specific lncRNAs and five WT-specific miRNAs for further analysis. The five miRNAs recognized above were mapped into the TargetScan, miRTarBase, and miRDB databases to identify their target mRNAs. The 150 mRNAs predicted by all three databases were considered target mRNAs candidates, and only 27 of them which were differentially expressed in WT tissues were identified as DEmRNAs for further analysis. Finally, 90 DElncRNAs, 5 DEmiRNAs, and 27 DEmRNAs enrolled the ceRNA network with 177 interactions (145 DElncRNA–DEmiRNA interactions and 32 DEmiRNA–DEmRNA interactions), constructed, and visualized with Cytoscape (Figure 6 and Table 2).

The rhombuses represent DElncRNAs, the squares represent DEmiRNAs, and the rounds represent DEmRNAs. Red represents upregulation and green represents downregulation.

### 3.6. Prognosis-Associated RNAs in the WT ceRNA Network

**Figure 7:**
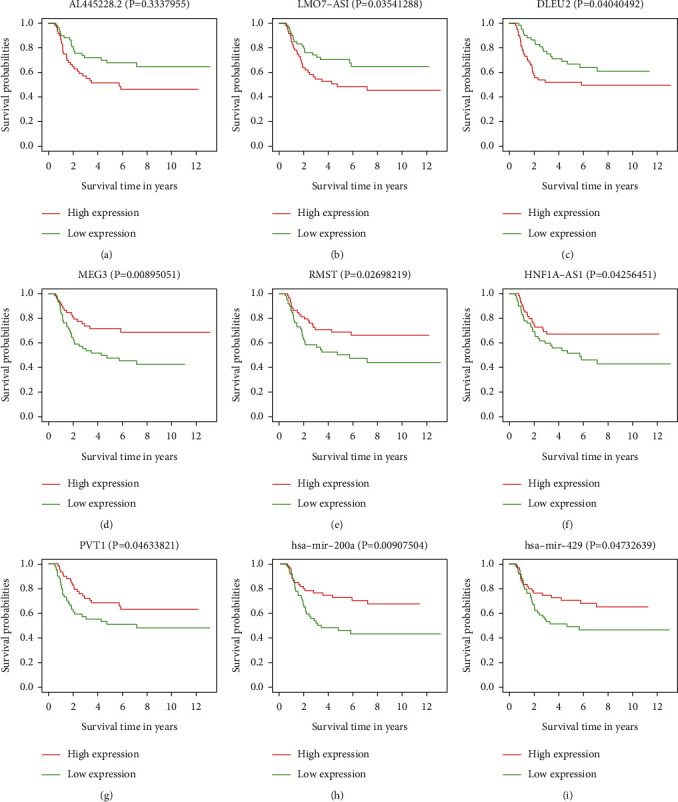
Kaplan–Meier survival curves for the prognosis-associated DERNAs. Kaplan–Meier survival curves for lncRNA (a) AL445228.2, (b) LMO7-AS1, (c) DLEU2, (d) MEG3, (e) RMST, (f) HNF1A-AS1 and (g) PVT1) and miRNAs (h) hsa-mir-429 and (i) hsa-mir-200a.

To identify the prognosis-associated RNAs, the Kaplan–Meier survival curve and log-rank test analysis of all the RNAs involved in the ceRNA network were performed. According to the results, 7 DElncRNAs and 2 DEmiRNAs were identified as prognosis-associated RNAs (*P* < 0.05, Figure 7).

Among these RNAs, the high expression of three risky lncRNAs including AL445228.2, LMO7-AS1 LMO7 antisense RNA (1), and DLEU2 (deleted in lymphocytic leukemia 2) was associated with shorter overall survival of WT patients (Figures 7(a) and 7(c)). The remaining four DElncRNAs (MEG3, maternally expressed gene 3; RMST, rhabdomyosarcoma 2-associated transcript; HNF1A-AS1, HNF1A antisense RNA 1; and PVT1, and plasmacytoma variant translocation 1) and two DEmiRNAs (hsa-mir-429 and hsa-mir-200a) appeared to be protective (Figures 7(d) and 7(i)).

In the figure, Kaplan–Meier survival curves for lncRNA (a) AL445228.2, (b) LMO7-AS1, (c) DLEU2, (d) MEG3, (e) RMST, (f) HNF1A-AS1, (g) PVT1 and miRNAs, (h) hsa-mir-429, and (i) hsa-mir-200a are shown.

## 4. Discussion

Several genes, including CTNNB1, WTX, WT1, and TP53, have been reported to be involved in the tumorigenesis and progression of WT [7–9]. However, the regulatory function of dysregulated RNAs (including lncRNAs, miRNAs, and mRNA) in WT remains elusive. Increasing evidence has revealed that dysregulated RNAs play important roles in many cancers [31–33]. miRNA profile changes in WT have been used as predictors of chemo responsiveness in WT blastema [34]. LINC00473 mediates the pathogenesis of WT by antagonizing the tumor suppressor hsa-mir-195 [35]. The elevated expression of FOXM1 (forkhead box protein M1) has been reported as a new prognostic biomarker that is associated with histology and prognosis of WT [36]. Therefore, identification of key RNAs is vital for understanding the pathogenesis and abnormal biological behavior of WT which may help to identify novel therapeutic targets.

First, we analyzed the microarray data and RNA-sequencing data from the GEO and TARGET databases. Then, 450 WT-specific mRNAs convergence in both databases was identified for bioinformatics analysis. Identify their potential associated cellular signaling pathways and functions. GO analysis revealed that the mRNAs were involved in the “organic anion transport” and “carboxylic acid biosynthetic process” biological processes terms. Furthermore, the mRNAs were demonstrated to serve a role in transmembrane transporter activity at the molecular function level. KEGG pathway analysis revealed that 12 mRNA were enriched in the “PPAR signaling pathway,” 10 were enriched in the “mineral absorption,” and 9 were enriched in “protein digestion and absorption.”

According to the MCODE analysis, a hub gene cluster with all the genes upregulated was identified. In line with the results of functional enrichment analysis of all WT-specific mRNAs, the hub genes were mostly enriched in the GO terms concerned with the “cell cycle” too, such as “nuclear division,” “organelle fission,” “mitotic nuclear division,” and “chromosome segregation.” These results suggested that drugs targeting the cell cycle may be effective in the treatment of WT. Expression validation of the hub genes was performed with Oncomine. The validation study showed that CENPF was unexpectedly downregulated, which may be due to the paucity of specimens in the Cutcliffe renal dataset and Yusenko renal dataset. Moreover, other hub genes were still upregulated in both datasets. Therefore, it can be hypothesized that abnormal regulation of the hub genes may be generally existed in WT and contribute to the tumorigenesis and progression of WT.

Subsequently, to comprehensively understand how dysregulated RNAs work in WT, we constructed a ceRNA with 90 DElncRNAs, 5 DEmiRNAs, 27 DEmRNAs, and 177 interactions. Nine of these RNAs, including AL445228.2, LMO7-AS1, DLEU2, MEG3, RMST, HNF1A-AS1, PVT1, hsa-mir-429, and hsa-mir-200a, were considered to be prognostic biomarkers as they were significantly associated with overall survival in WT patients. In line with the GO enrichment results, most of these RNAs were associated with tumor progression via the cell cycle.

Risky marker DLEU2 was reported to promote cancer cell proliferation and invasion in pancreatic cancer [37]. Protective lncRNA MEG3 was regarded as a novel tumor suppressor by inhibiting tumor cell proliferation in many cancers [38–40]. Protective lncRNA RMST (rhabdomyosarcoma 2-associated transcript) could enhance cell apoptosis in triple-negative breast cancer (TNBC) [41]. HNF1A-AS1 was considered to function as a regulator of cell proliferation and migration in esophageal adenocarcinoma [42] and lung cancer [43]. Still, there is WT-protective lncRNA which was a risky factor in other cancers. For example, PVT1 was reported to promote tumorigenesis in nonsmall cell lung cancer [44] and multidrug resistance in gastric cancer [45].

It is well-known that miRNA-regulated pathways are indispensable in studies of tumorigenesis [46, 47]. miRNAs are involved in the occurrence, development, incursions, and metastasis of tumors [48, 49]. In this study, among the five DEmiRNAs involved in the ceRNA network, two miRNAs (hsa-miR-200a and hsa-miR-429) were associated with the poor prognosis of WT patients. Both of them belong to the miR-200 family, which has been shown to be closely associated with carcinogenesis and progression, and potentially be important for the diagnosis and treatment of cancer [50]. hsa-mir-200a was reported to inhibit cell growth, migration, and invasion in meningioma [51] and nasopharyngeal carcinoma [52] and determines prognosis in colorectal cancer patients [53] and ovarian tumorigenesis [54]. As the has-mir-200 family is important for maintaining the epithelial phenotype [55], the dysregulation of hsa-mir-200a and hsa-mir-429 in WT patients may be associated with migration and invasion via the epithelial-mesenchymal transition.

## 5. Conclusions

In the present study, we successfully constructed a lncRNA-mediated ceRNA network with WT-specific lncRNAs and miRNAs/mRNAs convergence in both GEO and TARGET databases. The ceRNA network may provide novel insight into a better understanding of ceRNA regulation in WT. Furthermore, the ceRNA network and the key RNAs identified in this study may provide potential biomarkers for diagnosis and prognosis and improve clinical outcomes for children with WT.

## Figures and Tables

**Table 1:** The main characteristics of 125 Wilms tumor patients.

Clinicopathological characteristics	Patients (*N* = 125)	
	*n*	%
Age (years)		
≤5	77	61.60
5–10	41	32.80
≥10	7	5.60
Gender		
Male	56	44.80
Female	69	55.20
Histology classification of primary tumor		
FHWT	85	68.00
DAWT	40	32.00
First event		
Relapse	93	74.40
Progression	7	5.60
None	25	20.00
Stage		
I	16	12.80
II	52	41.60
III	40	32.00
IIIB	4	3.20
IIIB/V	1	0.80
IV	12	9.60

FHWT, favorable histology; DAWT, diffuse anaplasia.

**Table 2:** The DERNAs enrolled in the ceRNA network.

DElncRNAs	DEmiRNAs	DEmRNAs
KIAA0087 C22orf24 SHANK2-AS3 C20orf197 AC008738.1 C9orf106 AC022148.1 WDFY3-AS2 AC123768.1 AC092535.1 DSCR4 TMEM105 MIR22HG LINC00523 BX255923.1 LINC00501 PSORS1C3 AC008676.1 LINC00487 AC006305.1 UCA1 MEG3 AC104472.1 LINC00269 AL158151.1 DSCR4-IT1 GPC6-AS2 THRB-IT1 LINC00337 LINC00457 ZNF503-AS1 FAM138 B LINC00343 SHANK2-AS1 HCG15 PCGEM1 LINC00310 DNMBP-AS1 LMO7-AS1 HOTAIR AC011483.1 HCG22 LINC00242 ERVMER61-1 LINC00276 AL161645.1 DLEU2 NALCN-AS1 ZFY-AS1 LINC00472 LINC00460 LINC00284 GAS5 ZRANB2-AS1 LINC00494 LINC00484 EGOT GPC5-AS1 PRKX-AS1 AC016745.1 AL353626.1 AC097717.1 AL118511.1 TM4SF1-AS1 AC100832.2 HNF1A-AS1 AP000662.1 ADAMTS9-AS2 AC008781.1 AL353803.1 AC006238.1 GLYCTK-AS1 AC020704.1 HCG17 AL445228.2 NEAT1 SNHG6 LINC00535 AL139147.1 EGFLAM-AS4 PVT1 LINC00491 LNX1-AS1 PCAT1 GRM5-AS1 C8orf49 SNHG1 RMST MEG8 ITPK1-AS1	hsa-mir-204	IL11 PRRG4 IRF1 USP53 MACC1 ARHGAP29 THRB CDH2 ERMP1 SLC22A6 E2F3 TIAM1 HAS2 PARD6B TPD52L1 CCNE2 DENND5B ELAVL4 WASF3 JARID2 H2AFZ PTPRD CHRDL1 ELAVL2 ARAP2 STAT4 KLHL40
	hsa-mir-141	
	hsa-mir-200a	
	hsa-mir-429	
	hsa-mir-383	

## Data Availability

The datasets generated and analyzed during the current study are available in the GEO database (https://www.ncbi.nlm.nih.gov/geo/), the Oncomine (https://www.Oncomine.org), and the TARGET database (https://ocg.cancer.gov/programs/target).
